# Acidemia does not affect outcomes of patients with acute cardiogenic pulmonary edema treated with continuous positive airway pressure

**DOI:** 10.1186/cc9315

**Published:** 2010-11-01

**Authors:** Stefano Aliberti, Federico Piffer, Anna Maria Brambilla, Angelo A Bignamini, Valentina D Rosti, Tommaso Maraffi, Valter Monzani, Roberto Cosentini

**Affiliations:** 1 Dipartimento toraco-polmonare e cardio-circolatorio, University of Milan, IRCCS Fondazione Cà Granda Ospedale Maggiore Policlinico, via F. Sforza 35, 20122 Milan, Italy; 2 Emergency Medicine Department, IRCCS Fondazione Cà Granda Ospedale Maggiore Policlinico, via F. Sforza 35, 20122 Milan, Italy; 3 School of Specialization in Hospital Pharmacy, University of Milan, Via Colombo 71, 20133 Milan, Italy

## Abstract

**Introduction:**

A lack of data exists in the literature evaluating acidemia on admission as a favorable or negative prognostic factor in patients with acute cardiogenic pulmonary edema (ACPE) treated with non-invasive continuous positive airway pressure (CPAP). The objective of the present study was to investigate the impact of acidemia on admission on outcomes of ACPE patients treated with CPAP.

**Methods:**

This was a retrospective, observational study of consecutive patients admitted with a diagnosis of ACPE to the Emergency Department of IRCCS Fondazione Cà Granda Ospedale Maggiore Policlinico, Milan, Italy, between January 2003 and December 2006, treated with CPAP on admission. Two groups of patients were identified: subjects with acidemia (acidotic group), and those with a normal pH on admission (controls). The primary endpoint was clinical failure, defined as switch to bi-level ventilation, switch to endotracheal intubation or inhospital mortality.

**Results:**

Among the 378 patients enrolled, 290 (77%) were acidotic on admission. A total of 28 patients (9.7%) in the acidotic group and eight patients (9.1%) among controls experienced a clinical failure (odds ratio = 1.069, 95% confidence interval = 0.469 to 2.438, *P *= 0.875). Survival analysis indicates that, among acidotic patients, the time at which 50% of patients reached the 7.35 threshold was 173 minutes (95% confidence interval = 153 to 193). Neither acidemia (*P *= 0.205) nor the type of acidosis on admission (respiratory acidosis, *P *= 0.126; metabolic acidosis, *P *= 0.292; mixed acidosis, *P *= 0.397) affected clinical failure after adjustment for clinical and laboratory factors in a multivariable logistic regression model.

**Conclusions:**

Neither acidemia nor the type of acidosis on admission should be considered risk factors for adverse outcomes in ACPE patients treated with CPAP.

## Introduction

International guidelines suggest the use of non-invasive continuous positive airways pressure (CPAP) as first-line intervention in patients with acute cardiogenic pulmonary edema (ACPE) [1]. CPAP has proven to be easier to use, quicker to implement in clinical practice and to carry smaller associated costs in comparison with non-invasive ventilation (NIV) [2]. In light of these findings, CPAP has also been also used to treat ACPE patients outside the intensive care unit or the Emergency Department, as in the general ward or during prehospital care [3].

The rate of mortality in ACPE patients treated with CPAP is reported to be up to 13% [4,5]. Therefore, it is crucial for healthcare providers to identify risk factors for failure of CPAP treatment, in order to better allocate medical resources and improve clinical outcomes of ACPE patients.

Severity of acidemia on admission, as well as lack of improvement of respiratory acidosis during the first few hours of NIV, have emerged as important predictors of failure in patients suffering of hypercapnic respiratory failure [6-8]. Acidemia on admission has been also shown to predict NIV failure a few days after its initial application in patients who have previously experienced an initial improvement of clinical status and blood gas values [9]. In clinical practice, acidotic patients with ACPE are commonly considered more severe in comparison with nonacidotic patients. In view of this consideration, the largest clinical trial that has evaluated CPAP and NIV in ACPE patients enrolled acidotic patients [10].

On the contrary, acidemia has not been identified as a predictor of NIV failure in patients with hypoxemic respiratory failure [5,11]. Conflicting data exist in the literature alternatively considering respiratory acidosis a favorable or a negative prognostic factor in ACPE patients. Particularly, ACPE patients who suffered respiratory acidosis on admission were identified as those exhibiting a better response to CPAP treatment [12].

To define the impact of acidemia on clinical outcomes of ACPE patients treated with CPAP, the present study has the following objectives: to compare outcomes and physiological measurements of patients with acidemia versus those with normal pH values on admission; and to evaluate outcomes and physiological measurements of patients with different types of acidosis on admission.

## Materials and methods

### Setting and subjects

This was a retrospective, observational study of consecutive patients admitted with a diagnosis of ACPE to the Emergency Department of IRCCS Fondazione Ca' Granda Ospedale Maggiore Policlinico, Milan, Italy between January 2003 and December 2006.

Adult patients who satisfied the criteria for ACPE and who were treated with CPAP on admission were enrolled in the study. Patients with alkalemia on admission were excluded.

The diagnosis of ACPE was established on the basis of medical history (acute severe dyspnea) and typical physical findings (widespread pulmonary rales), with chest radiography confirming pulmonary vascular congestion. Criteria for application of CPAP included at least one of the following: severe acute respiratory failure (PaO_2_/FiO_2 _ratio <300); respiratory rate exceeding 30 breaths/minute or use of accessory respiratory muscles or paradoxical abdominal motion; and respiratory acidosis (pH <7.350, PaCO_2 _≥45 mmHg).

All patients enrolled in the study underwent high-flow CPAP (90 to 140 l/minute; VitalSigns Inc., Totowa, NJ, USA) as the first choice of treatment, in addition to oxygen and standard medical treatment. Interfaces used were a facemask (VitalSigns Inc.) or a helmet (StarMed, Mirandola, Italy) with a positive end-expiratory pressure (PEEP) valve (VitalSigns Inc.). CPAP was not applied in ACPE patients if any among the following findings was present: immediate need for endotracheal intubation; impairment of consciousness (Kelly scale >4) [13]; and hemodynamic instability (systolic blood pressure <90 mmHg). Criteria for discontinuation from CPAP included all of the following: absence of respiratory distress; respiratory rate <25 beats/minute; hemodynamic stability; pH >7.35; and PaO_2_/FiO_2 _ratio >300 or oxygen saturation ≥95%.

Criteria to switch from CPAP to bi-level ventilation were a lack of improvement or a worsening of ventilation and/or gas exchange at a blood gas examination performed 30 minutes/1 hour after initiation of CPAP treatment, in the absence of criteria for endotracheal intubation (ETI). Criteria for ETI were at least one among the following: impairment of consciousness; hemodynamic instability (systolic blood pressure <90 mmHg); cardiac and/or respiratory arrest; and a lack of improvement or a worsening of ventilation and/or gas exchange at a blood gas examination performed 1 hour after initiation of bi-level treatment.

The above criteria for the application of CPAP in ACPE patients as well as the protocol of medical treatment were applied according to local standard operating procedures. Each patient received medical treatment according to the local standard of care: intravenous furosemide 40 to 100 mg based on fluid retention (or at least doubling the dose at home) targeted on the urinary output; intravenous isosorbide dinitrate on continuous infusion starting at 1 mg/hour up to 10 mg/hour; intravenous morphine up to 4 mg and vasopressors in case of hypotension. No subjects receiving invasive or non-invasive pressure support ventilation before CPAP treatment were included in the study.

### Study design

Records of all the enrolled patients were carefully reviewed. Data on admission, before and during CPAP treatment, and during hospitalization were collected, and included the following: demographic information and past medical history; clinical characteristics; laboratory evaluation performed on arterial sample; and information needed to derive the Simplified Acute Physiology Score II [14]. Arterial blood gas evaluation on admission was considered for those samples obtained within 15 minutes from admission to the hospital, based on local standard operating procedures. A group of investigators of the Emergency Department, Fondazione Ca' Granda, Milan, Italy validated the quality of data by checking for discrepancies and inconsistencies before cases were entered into a database. The Institutional Review Board of the IRCCS Fondazione Ca' Granda Ospedale Maggiore Policlinico, Milan approved the study. The study was in compliance with the Helsinki Declaration; informed consent was waived by the Institutional Review Board.

### Study definitions

The normal pH range was considered 7.35 to 7.45. Alkalemia was considered if the pH value on admission was more than 7.45. Acidemia was considered if the pH value on admission was less than 7.35. Respiratory acidosis was considered when acidemia was identified with PaCO_2 _≥45 mmHg and bicarbonates (HCO_3_^-^) ≥22 mmol/l. Metabolic acidosis was considered when acidemia was identified with PaCO_2 _<45 mmHg and HCO_3_^- ^<22 mmol/l. Mixed acidosis was considered when acidemia was identified with PaCO_2 _≥45 mmHg and HCO_3_^- ^<22 mmol/l.

### Study groups

Patients with ACPE treated with CPAP were divided into two groups according to the pH value on admission: subjects with acidemia (acidotic group), and those with a normal pH (controls). Among patients of the acidotic group, three subgroups were identified according to PaCO_2 _and HCO_3_^- ^values: patients with respiratory acidosis, patients with metabolic acidosis, and patients with mixed acidosis.

### Endpoints

The primary endpoint was clinical failure, defined as at least one among: a switch to non-invasive bi-level ventilation, a switch to ETI, and inhospital mortality.

A switch to bi-level ventilation was applied when both blood gas values were unchanged/worsened with CPAP and criteria for ETI were not fulfilled. ETI was performed according to our local standard operating procedures. Inhospital mortality was defined as death by any cause occurring during hospitalization. ACPE-related mortality was defined as death occurring during the episode of ACPE. Late mortality was defined as death occurring after the resolution of the episode of ACPE. Our local standard operating procedures define an episode of ACPE as being resolved when all the criteria for discontinuation of CPAP mentioned above are reached.

The secondary endpoint was the length of stay in the hospital. This length of stay was calculated as the number of days from the date of admission to the date of discharge, and was censored at 14 days in an effort to capture only the ACPE-related length of stay in the hospital.

### Statistical analysis

All data were statistically analyzed with SPSS for Windows (version 14.0; SPSS Inc., Chicago, IL, USA). Descriptive statistics are reported as the mean with standard deviation or counts and proportions as appropriate. Patient characteristics were compared between groups. Summary statistics for all continuous explanatory variables are presented as means with differences between groups compared by independent *t *test. Categorical explanatory variables are summarized as percentages with differences between groups analyzed using the chi-square test or the Fisher exact test where appropriate. The time to event was analyzed by Kaplan-Meier survival analysis. The association between clinical failure and acidemia on admission was analyzed using multiple logistic regression. All explanatory variables considered of clinical relevance and those previously found to be significantly associated with mortality in ACPE patients treated with CPAP were incorporated into the model [5]. The time course of continuous variables was analyzed by repeated-measures analysis of variance after replacing the missing values with the last observation carried forward technique. *P *< 0.05 was considered statistically significant.

## Results

### Acidotic population

Among the 419 ACPE patients treated with CPAP who were enrolled during the study period, the pH value within 15 minutes from admission was not available in 23 patients, while 18 patients were excluded because of alkalemia on admission. The final study population accounted for 378 patients: 290 (77%) were acidotic on admission (acidotic group), while 88 were controls. Baseline characteristics and the CPAP setting of the acidotic group and controls are summarized in Table 1.

**Table 1:** Baseline characteristics on admission and before continuous positive airway pressure treatment

Variable	Acidotic group (*n *= 290)	Controls (*n *= 88)	*P *value
Demographics			
Male	143 (49)	36 (41)	0.167^a^
Age (years)	80 ± 10 (*n *= 290)	81 ± 9.5 (*n *= 88)	0.360^b^
Comorbidities			
Chronic obstructive pulmonary disease	84 (29)	17/86 (20)	0.091^a^
Essential hypertension	162 (56)	46/86 (54)	0.697^a^
Diabetes mellitus	72 (25)	19/86 (22)	0.603^a^
Congestive heart failure	165 (57)	51/86 (59)	0.692^a^
Chronic renal failure	76 (26)	13/86 (15)	0.034^a^
Severity of the disease			
Simplified Physiologic Acute Score II	42 ± 6.7 (*n *= 258)	40 ± 8.1 (*n *= 74)	0.014^b^
Physical findings			
Systolic blood pressure (mmHg)	173 ± 30 (*n *= 286)	170 ± 31 (*n *= 87)	0.328^b^
Diastolic blood pressure (mmHg)	99 ± 20 (*n *= 283)	97 ± 19 (*n *= 87)	0.391^b^
Systolic <140 mmHg and diastolic <90 mmHg	32 (11)	9 (10)	0.802^b^
Heart rate (beats/minute)	116 ± 22 (*n *= 283)	121 ± 22 (*n *= 87)	0.163^b^
Heart rate >100 beats/minute	197/283 (70)	53/87 (61)	0.130^a^
Respiratory rate (breaths/minute)	41 ± 6.1 (*n *= 175)	39 ± 6.9 (*n *= 64)	0.016^b^
Respiratory rate ≥40 breaths/minute	120/175 (69)	30/64 (47)	0.002^a^
Arterial blood gas analysis			
pH	7.22 ± 0.09 (*n *= 290)	7.39 ± 0.03 (*n *= 88)	Not applicable
PaCO_2 _(mmHg)	53 ±16 (*n *= 290)	36 ±6.6 (*n *= 88)	<0.001^b^
Bicarbonates (mmol/l)	22 ± 5.3 (*n *= 288)	22 ± 3.8 (*n *= 88)	0.330^b^
PaO_2_/FiO_2 _ratio	178 ± 93 (*n *= 283)	222 ± 82 (*n *= 87)	<0.001^b^
PaO_2_/FiO_2 _ratio <200	184/283 (65)	32/87 (37)	<0.001^a^
Acute myocardial infarction on admission	43 (15)	14 (16)	0.804^a^
CPAP setting			
Initial FiO_2 _(%)	49.7 ± 12.1 (*n *= 288)	48.6 ± 11.4 (*n *= 88)	0.421^b^
Initial PEEP (cmH_2_O)	9.7 ± 2.0 (*n *= 290)	9.7 ± 1.3 (*n *= 88)	0.927^b^
Device			
Face mask	38 (19)	15 (24)	0.475^a^
Helmet	157 (81)	48 (76)	
Information not available	95	29	

Demographics, comorbidities, severity of the disease, clinical and laboratory findings on admission and before continuous positive airway pressure (CPAP) treatment of the study population, according to the presence or absence of acidemia on admission. Data presented as number (%) or mean ± standard deviation. PaCO_2_, partial pressure of carbon dioxide in arterial blood; PaO_2_/FiO_2_, partial pressure of oxygen in arterial blood/inspired oxygen fraction; PEEP, positive end-expiratory pressure. ^a^Chi-square test. ^b^Unpaired *t *test.

The mean ± standard deviation duration of CPAP treatment was 318 ± 485 minutes and 262 ± 198 minutes in the acidotic group and controls, respectively (*P *= 0.289). The mean ± standard deviation FiO_2 _during CPAP was 48 ± 11% and 47 ± 9% in the acidotic group and in controls, respectively (*P *= 0.219). The mean ± standard deviation PEEP during CPAP was 8.1 ± 1.7 cmH_2_O and 7.9 ± 1.4 cmH_2_O in the acidotic group and in controls, respectively (*P *= 0.229).

A total of 28 patients (9.7%) in the acidotic group and eight patients (9.1%) among controls experienced a clinical failure (odds ratio = 1.069; 95% confidence interval = 0.469 to 2.438; *P *= 0.875) (see Table 2). Acidemia on admission did not affect clinical failure after adjustment for age, history of acute myocardial infarction, hypocapnia, normotension and PaO_2_/FiO_2 _ratio in a multivariable logistic regression model (*P *= 0.205).

**Table 2:** Clinical endpoints of the study population, according to presence or absence of acidemia on admission

Variable	Acidotic group (*n *= 290, 77%)	Controls (*n *= 88, 23%)	*P *value (chi-square test)
Clinical failure	28 (9.7)	8 (9.1)	0.875
Change to bi-level	5 (1.7)	0 (0)	0.215
Change to intubation	6 (2.1)	0 (0)	0.174
ACPE-related mortality^a^	6 (2.1)	1 (1.1)	0.484
Late mortality^b^	17 (6.0)	7 (8.1)	0.488
In-hospital mortality^b^	23 (8.2)	8 (9.3)	0.738
Length of hospital stay (days)	11 ± 6.9	11 ± 6.3	0.617

Data presented as number (%) or mean ± standard deviation. ACPE, acute cardiogenic pulmonary edema. ^a^Two patients censored as by day 1. ^b^Ten patients censored as by day 1.

**Figure 1:**
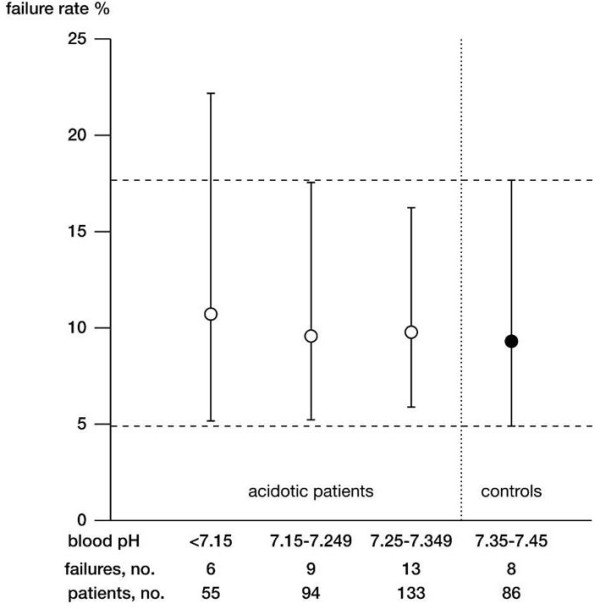
**Clinical failure rate of the study population by pH value on admission**. The 95% confidence intervals of the control group are depicted with dashed horizontal lines.

**Figure 2:**
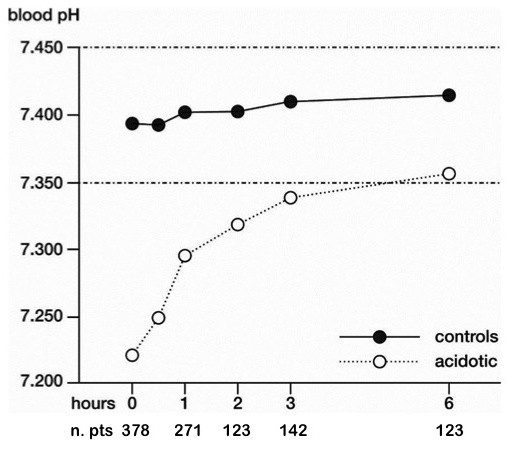
**Time course of pH during continuous positive airways pressure treatment**. The time course of mean arterial blood pH during continuous positive airways pressure treatment in the acidotic group and in controls. Adjusted for age and sex; missing data replaced with the last observation carried forward technique.

**Figure 3:**
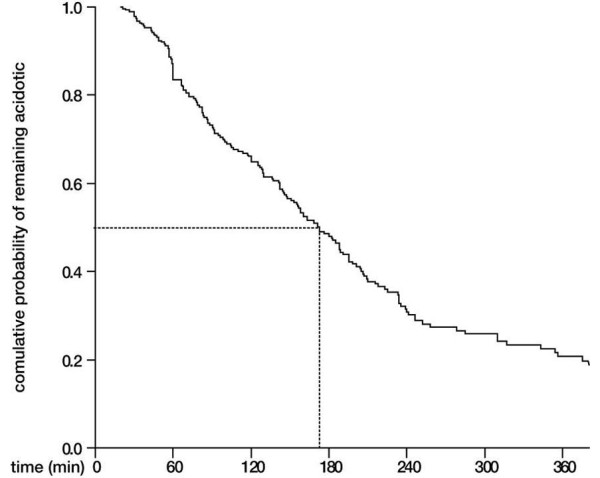
**Survival analysis of time to pH ≥7.350 among acidotic patients**. Dotted lines indicate the time at which 50% of the sample reached the threshold pH (173 minutes).

The crude proportion of clinical failure in the study population is presented in Figure 1, split by pH value on admission. The 95% confidence interval of the controls group included the point estimate and most of the confidence intervals of the other groups. Figure 2 shows the time course of the mean arterial pH in the study population. Survival analysis indicates that, among acidotic patients, the time at which 50% of patients reached the 7.35 threshold was 173 minutes (95% confidence interval = 153 to 193) (see Figure 3).

### Respiratory, metabolic and mixed acidotic populations

Among the 290 acidotic patients, 13 could not be further classified. Among the other 277 patients, 122 (44%) showed a respiratory acidosis, 89 (32%) a metabolic acidosis, and 66 (24%) a mixed acidosis on admission. The baseline characteristics and CPAP setting of the acidotic population are summarized in the supplemental digital content in Additional file 1, according to the type of acidemia on admission.

A total of 12 patients (10%) with respiratory acidosis, 11 patients (13%) with metabolic acidosis, four patients (6.2%) with mixed acidosis and eight controls (9.3%) experienced clinical failure (*P *= 0.613) (see Table 3). The type of acidosis on admission did not affect clinical failure after adjustment for age, history of acute myocardial infarction, hypocapnia, normotension and PaO_2_/FiO_2 _ratio in a multivariable logistic regression model (respiratory acidosis, *P *= 0.126; metabolic acidosis, *P *= 0.292; mixed acidosis *P *= 0.397).

**Table 3:** Clinical endpoints of the study population based on type of acidosis on admission

Variable	Respiratory acidosis (*n *= 122)	Metabolic acidosis (*n *= 89)	Mixed acidosis (*n *= 66)	Controls (*n *= 88)	*P *value (chi-square test)
Clinical failure	12 (10)	11 (13)	4 (6.2)	8 (9.3)	0.613
Change to bi-level	5 (4.1)	0 (0)	0 (0)	0 (0)	0.018
Change to intubation	1 (0.8)	2 (2.2)	2 (3.0)	0 (0)	0.341
ACPE-related mortality	1 (0.8)	4 (4.5)	1 (1.5)	1 (1.1)	0.237
Late mortality	8 (6.8)	7 (8)	2 (3.1)	7 (8.1)	0.595
In-hospital mortality	9 (7.6) (CI, 4.1 to 14.1)	11 (12.6) (CI, 7.4 to 21.7)	3 (4.6) (CI, 1.6 to 13.1)	8 (9.3) (CI, 4.9 to 17.7)	0.351
Length of hospital stay (days)	11 ± 7	11 ± 9	10 ± 5	13 ± 22	0.582^a^

Data presented as number (%) or mean ± standard deviation. ACPE: acute cardiogenic pulmonary edema; CI, 95% confidence interval. ^a^One-way analysis of variance.

**Figure 4:**
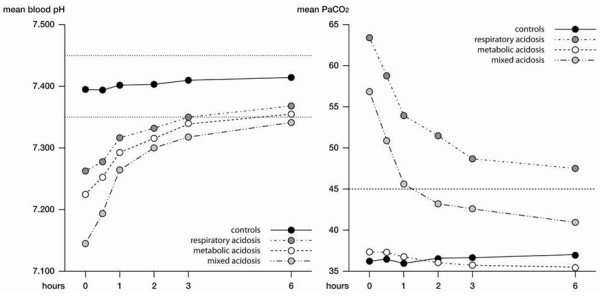
**Time course of pH and PaCO_2 _during continuous positive airways pressure treatment**. Time course of pH and partial pressure of carbon dioxide in arterial blood (PaCO_2_) during continuous positive airways pressure treatment in the controls and in the acidotic group according to the diagnosis (after replacing the missing values according to the last observation carried forward technique and after adjustment for age, sex and systolic blood pressure).

The time course of both pH and PaCO_2 _values during CPAP treatment in the acidotic groups, based on diagnosis at admission, as well as in controls is depicted in Figure 4, after replacing the missing values according to the last observation carried forward technique and after adjustment for age, sex and systolic blood pressure. An increase in pH values was detected in all groups of patients regardless of the type of acidosis, while a decrease in PaCO_2 _values was observed in mixed and respiratory acidosis patients.

## Discussion

The present study indicates that acidemia on admission is not a risk factor for adverse outcomes in ACPE patients treated with CPAP. Furthermore, not even the type of acidosis on admission - respiratory, metabolic or mixed - impacts clinical outcomes of ACPE patients treated with CPAP.

Among our cohort of ACPE patients treated with CPAP, more than three-quarters were acidotic on admission. Our acidotic patients showed similar clinical and laboratory characteristics on admission in comparison with the 346 ACPE acidotic patients treated with CPAP enrolled in the randomized controlled trial by Gray and coworkers [10]. The present study, however, reported lower ACPE-related, late and inhospital mortality rates than those reported in that trial. Possible explanations could be found in the CPAP setting (ventilator with a low initial PEEP), as well as the length of treatment used in the study by Gray and colleagues. In this last study the mean duration of CPAP treatment was 2 to 3 hours. We showed that, while CPAP treatment in acidotic ACPE patients did actually bring 50% of patients to a pH value above 7.35 within 3 (2.5 to 6) hours, the treatment nevertheless had to be protracted for at least 6 hours before the mean pH crossed the threshold of 7.35.

We found that acidemia on admission is not a risk factor for failure in ACPE patients treated with CPAP. To date, no studies have evaluated the impact of the degree of acidemia on admission on outcomes of ACPE patients treated with CPAP. We found that the degree of acidemia on admission seems not to be associated with failure. This surprising finding could be explained by the rapidity of the resolution of acidemia in our ACPE patients during CPAP treatment. The increase of pH seems to be particularly crucial during the first hours of CPAP treatment, and thus the pH evaluation during this timeframe would be a better marker of prognosis rather than the single value of pH on admission.

One of the main implications of these findings is that acidotic patients with ACPE undergoing CPAP treatment should not be considered more severe than those with a normal pH value on admission. On the other hand, other clinical and laboratory factors should be considered in the severity assessment of the ACPE population treated with CPAP, such as advanced age, normal-to-low blood pressure, hypocapnia, or severe alteration of gas exchange [5].

We found that the type of acidosis on admission (respiratory, metabolic as well as mixed acidosis) does not significantly modify the clinical outcomes in ACPE patients treated with CPAP. ACPE patients with respiratory acidosis on admission undergoing CPAP treatment seem to benefit from this technique. In our study, we found a decrease in PaCO_2 _levels with a consequent recovery of pH values during CPAP treatment in respiratory acidotic patients. An explanation for this finding could be identified in the rationale of the increase of PaCO_2 _during an episode of ACPE. The etiology of hypoventilation as a sign of pump failure is twofold. On the one hand, such as among patients with acute exacerbation of chronic bronchitis, hypercapnia, often acute on chronic, occurs due to an increased load of the respiratory system and reduced muscular force related to the presence of bronchial obstruction and intrinsic PEEP. On the other hand, such as among patients with ACPE without chronic pump failure, the acute hypoventilation is strictly related to decreased compliance due to parenchymal causes (interstitial/alveolar flooding), and is thus easily reversed by the alveolar recruitment induced by PEEP. Our findings support data from Bellone and colleagues, who in an elegant randomized controlled trial showed that CPAP could be used in acidotic patients [11]. Based on these data, excluding *a priori *the use of CPAP in ACPE patients who present respiratory acidosis on admission could not be justified.

We also found an improvement in pH values in ACPE patients with metabolic acidosis on admission undergoing CPAP treatment. This interesting finding could be explained in light of beneficial effects of the application of PEEP on the heart and hemodynamics, as well as tissue perfusion in patients with ACPE. The most severe ACPE patients treated with CPAP in our population were those with mixed acidosis on admission who showed the lowest pH values, mainly because of a double effect on both the respiratory and metabolic systems. During CPAP treatment, we found these patients to have a quicker increase of pH values in comparison with the other acidotic patients, in light of the double action of CPAP on both respiratory mechanics and hemodynamics.

In view of its retrospective design, a weakness of our study could be a deficiency in accurately collecting some history and clinical information. To our knowledge, the present study is the first to evaluate the impact of different acidosis patterns on admission in ACPE patients treated with CPAP. This study is strengthened by a large sample size of consecutive ACPE patients. Moreover, our findings are representative of an unselected population, and our conclusions can thus be easily generalized.

## Conclusions

Neither acidemia nor the type of acidosis on admission should be considered a risk factor for adverse outcomes in ACPE patients treated with CPAP. Furthermore, we suggest that nonacidotic patients should be included in future clinical trials, being at least as severe as the acidotic population.

## Key messages

• Acidemia on admission is not a risk factor for adverse outcomes in patients with ACPE treated with CPAP.

• The type of acidosis on admission - respiratory, metabolic or mixed - does not impact clinical outcomes of ACPE patients treated with CPAP.

## Abbreviations

ACPE: acute cardiogenic pulmonary edema; CPAP: continuous positive airways pressure; ETI: endotracheal intubation; HCO_3_^-^: bicarbonates; NIV: non-invasive ventilation; PaCO_2_: partial pressure of carbon dioxide in arterial blood; PaO_2_/FiO_2_: partial pressure of oxygen in arterial blood/inspired oxygen fraction; PEEP: positive end-expiratory pressure.

## Competing interests

The authors declare that they have no competing interests.

## Authors' contributions

SA contributed to the conception and design of the study, as well as the acquisition, analysis and interpretation of data; he was involved in drafting the manuscript and revising it critically for important intellectual content. RC and AMB contributed to the conception and design of the study, the analysis and interpretation of data; they were involved in revising the manuscript. AAB contributed to the conception and design, analysis and interpretation of data; he was involved in revising the manuscript. FP, TM and VDR contributed to the acquisition, analysis and interpretation of the data; they were involved in revising the manuscript critically. VM revised the manuscript. All authors read and approved the final manuscript.

## Supplementary Material

Additional file 1 **The acidotic population**. A Word table presenting demographics, comorbidities, severity of the disease, clinical and laboratory findings on admission and before CPAP treatment of the acidotic population, according to the type of acidemia on admission. Click here for file
