# Supplementary material for: Acidemia does not affect outcomes of patients with acute cardiogenic pulmonary edema treated with continuous positive airway pressure
Source: Crit Care. 2010 Nov 1;14(6):R196. doi: 10.1186/cc9315 (PMC3220020; doi:10.1186/cc9315)
Supplement: Additional file 1 — The acidotic population. A Word table presenting demographics, comorbidities, severity of the disease, clinical and laboratory findings on admission and before CPAP treatment of the acidotic population, according to the type of acidemia on admission. [file cc9315-S1.DOC]

**Table Online resource. Demographics, comorbidities, severity of the disease, clinical and laboratory findings on admission and before CPAP treatment of the acidotic population, according to the type of acidemia on admission.**

| **Variable** |  | **Respiratory acidosis** | **Metabolic acidosis** | **Mixed acidosis** | **p value** |
| --- | --- | --- | --- | --- | --- |
| Total monitored patients, n. |  | 122 | 89 | 66 |  |
| **Demographics** |  |  |  |  |  |
| Male | n/N (%) | 58/122 (48) | 47/89 (53) | 34/66 (52) | 0.729[1] |
| Age, years | mean ± SD (N) | 80 ± 10 (122) | 78 ± 12 (89) | 79 ± 8 (66) | 0.445[2] |
| **Comorbidities** |  |  |  |  |  |
| Chronic obstructive pulmonary disease | n/N (%) | 45/122 (37) | 18/89 (20) | 18/66 (27) | 0.029[1] |
| Essential hypertension | n/N (%) | 74/122 (61) | 49/89 (55) | 33/66 (50) | 0.357[1] |
| Diabetes mellitus | n/N (%) | 33/122 (27) | 22/89 (25) | 14/66 (21) | 0.676[1] |
| Congestive heart failure | n/N (%) | 64/122 (53) | 50/89 (56) | 42/66 (64) | 0.337[1] |
| Chronic renal failure | n/N (%) | 18/122 (15) | 35/89 (39) | 19/66 (29) | <0.001[1] |
| **Severity of the disease** |  |  |  |  |  |
| SAPS II | mean ± SD (N) | 40±6.3 (107) | 44±6.9 (79) | 44±5.8 (60) | <0.001[2] |
| **Physical findings** |  |  |  |  |  |
| Systolic BP, mmHg | mean ± SD (N) | 177±32 (122) | 166±30 (86) | 175±27 (65) | 0.036[2] |
| Diastolic BP, mmHg | mean ± SD (N) | 101±20 (121) | 95±20 (86) | 102±19 (63) | 0.062[2] |
| Systolic BP <140 mmHg and diastolic BP <90 mmHg | n/N (%) | 14/121 (12) | 12/86 (14) | 5/63 (7.9) | 0.523[1] |
| Heart rate, beats/min | mean ± SD (N) | 112±19 (120) | 114±24 (86) | 125±18 (64) | <0.001[2] |
| Heart rate >100 beats/min | n/N (%) | 75/120 (63) | 54/86 (63) | 57/64 (89) | <0.001[1] |
| Respiratory rate, breaths/min | mean ± SD (N) | 40±5 (74) | 42±7 (48) | 42±6.8 (41) | 0.464[2] |
| Respiratory rate ≥40 breaths/min | n/N (%) | 48/74 (65) | 30/48 (63) | 33/41 (81) | 0.139[1] |
| **Arterial Blood gas analysis** |  |  |  |  |  |
| pH | mean ± SD (N) | 7.23±0.08 (122) | 7.26±0.08 (89) | 7.15±0.09 (66) | 0.000[2] |
| PaCO_2_, mmHg | mean ± SD (N) | 63±14 (122) | 37±6 (89) | 57±12 (66) | 0.000[2] |
| Bicarbonates, mmol/L | mean ± SD (N) | 26±3.9 (122) | 17±3.5 (89) | 19±2.3 (66) | 0.000[2] |
| PaO_2_/FiO_2_  ratio | mean ± SD (N) | 178±87 (121) | 186±106 (86) | 160±87 (64) | 0.258[2] |
| PaO_2_/FiO_2_  ratio <200 | n/N (%) | 79/121 (65) | 49/86 (57) | 50/64 (78) | 0.026[1] |
| **AMI on admission** | n/N (%) | 15/122 (12) | 17/89 (19) | 10/66 (15) | 0.396[1] |
| **CPAP Setting** |  |  |  |  |  |
| Initial FiO_2_, % | mean ± SD (N) | 47±9 (122) | 53±13 (88) | 51±14 (65) | <0.001[2] |
| Initial PEEP, cmH_2_O | mean ± SD (N) | 9.5±1.9 (122) | 9.8±2.3 (89) | 9.9±1.8 (66) | 0.411[2] |
| Device Face-mask | n. (%) | 18 (22) | 4 (7) | 13 (29) | 0.042[1] |
| Helmet | n. (%) | 62 (78) | 56 (93) | 31 (71) |  |
| Information N/A | n. (%) | 42 | 29 | 22 |  |

Footnotes: N: number; SD: standard deviation; SAPS II: Simplified Physiologic Acute Score II; BP: Blood Pressure. AMI: acute myocardial infarction; CPAP: continuous positive airway pressure; FiO_2_: fraction of inspired oxygen; PEEP: positive end-expiratory pressure; N/A: not available. [1] chi square test; [2] one-way Analysis of Variance
